# Liposomal vaccine formulations as prophylactic agents: design considerations for modern vaccines

**DOI:** 10.1186/s12951-017-0319-9

**Published:** 2017-11-17

**Authors:** Luis O. De Serrano, David J. Burkhart

**Affiliations:** Department of Biomedical & Pharmaceutical Sciences and Center for Translational Medicine, University of Montana, 32 Campus Drive, Missoula, MT 59812 USA

**Keywords:** Liposomes, Vaccine formulations, CLR, TLR, Viral infections, Bacterial infections, Parasites

## Abstract

Vaccinology is one of the most important cornerstones in modern medicine, providing better quality of life. The human immune system is composed of innate and adaptive immune processes that interplay when infection occurs. Innate immunity relies on pathogen-associated molecular patterns which are recognized by pathogen recognition receptors localized in antigen presenting cells. After antigen processing and presentation, CD4^+^ T cell polarization occurs, further leading to B cell and CD8^+^ activation and humoral and cell-mediated adaptive immune responses. Liposomes are being employed as vaccine technologies and their design is of importance to ensure proper immune responses. Physicochemical parameters like liposome size, charge, lamellarity and bilayer fluidity must be completely understood to ensure optimal vaccine stability and efficacy. Liposomal vaccines can be developed to target specific immune cell types for the induction of certain immune responses. In this review, we will present promising liposomal vaccine approaches for the treatment of important viral, bacterial, fungal and parasitic infections (including tuberculosis, TB). Cationic liposomes are the most studied liposome types due to their enhanced interaction with the negatively charged immune cells. Thus, a special section on the cationic lipid dimethyldioctadecylammonium and TB is also presented.

## Background

Vaccination is one of the most significant developments in modern science, improving the treatment and controlling the spread of diseases across communities. For formulation scientists and immunologists, there has been an interest in the development of liposomal vaccines for their prophylactic uses in infections (of viral, bacterial, fungal or parasitic origin) [1–3]. To develop safe and effective liposome-based vaccines, scientists should take into consideration several interconnected principles: (1) the design-dependent function of the liposomes (2) the characteristics of liposome-cell interactions when vaccine administration occurs and (3) the specific cell receptor and signaling involved once liposomal vaccines are administered. Each of these principles will affect vaccine efficacy and its potential development from bench to bedside applications. These three principles are also the basis for developing excellent subunit vaccine strategies, which have been of important interest for vaccine scientists for several years [4–7]. To comprehend how the potential liposomal vaccine might work in the host we must understand the immune responses involved in receptor signaling. The field of immunology plays a significant role that will help determine the utilization of vaccinology to the advantage of the patient by developing adequate prophylactic treatment approaches.

Immunologists investigate how our bodies defend themselves from pathogens by determining and describing the signaling mechanisms involved. Basically, when infection occurs, the innate arm of the immune system responds through recognition of distinct molecules on or in pathogens termed ‘pathogen associated molecular patterns (PAMPs)’. These PAMPs differ from host markers and as such are recognized by the first line of defense in the immune system, antigen presenting cells (APCs) such as dendritic cells (DCs), macrophages and neutrophils. The PAMPs are recognized by pattern recognition receptors (PRRs) on the surface and in endosomes of APCs [8–11]. Vertebrates developed the adaptive immune system as a second line of defense that could ‘remember’ pathogens and fight back upon re-challenge. It is composed of cells such as T and B cells that employ de novo synthesized antigen-specific receptors (T- and B cell receptors, TCRs and BCRs). TCR and BCRs are able to recognize pathogen-specific antigens when presented complexed with major histocompatibility complexes (MHC) on the surface of an APC. But polarization of the T or B cells to ensure it acts appropriately against the pathogen, relies on signals provided by the APC (such as co-stimulatory molecules and precise cytokines) in response to the priming by PAMPs. These critical signals lead to differentiation of effector and eventually memory cells that are critical for driving the immune response against future infections with the same pathogen. While innate immune responses are broadly applicable and adaptive immune responses are antigen-specific, the two arms of the immune system work in close concert to mount an effective response to clear the pathogen.

The development of PRRs by the immune system represent a significant advancement in the fight for survival of the host. Therefore, an important question arises to that end: what type of PRRs have been discovered so far and which ligands (or PAMPs) do they tend to recognize? Some PRRs are secreted to the extracellular milieu and participate in pathogen opsonization. However, most PRRs are transmembrane (like C-type lectin and Toll-like receptors; CLRs and TLRs, respectively) or cytosolic (retinoic acid-inducible gene I and nucleotide-binding domain and leucine-rich repeat containing receptors; RLRs and NLRs, respectively). For the purposes of this review, we will discuss the uses of liposomes in vaccines that target CLRs and TLRs. The TLRs identified so far amount to 10 in humans, each one recognizing specific PAMPs from microbial pathogens (Table 1) [10–12]. TLR 10 has been recently described as a modulatory receptor; however, no known ligand has been linked to it [13]. Therefore, further research should be done on TLR 10 to uncover the ligand involved in innate immune responses.

**Table 1:** Pathogen-associated molecular patters (PAMPs) recognized by specific TLRs

Pathogen-associated molecular pattern (PAMP)	Microorganism or classification	TLRs
Lipoproteins	Bacteria	TLR 1
Peptidoglycan and lipoteichoic acid	Gram positive bacteria	TLR 2
β-glucans	Fungi	
dsRNA	Double-stranded and negative-stranded viruses	TLR 3
Lipopolysacharide (LPS)	Gram negative bacteria	TLR 4
Flagelin	Bacteria	TLR 5
Profilin	*Toxoplasma gondii*	
Lipoproteins	*Mycoplasma*	TLR 6
Imidazoquinolines and ssRNA	Single-stranded viruses	TLR 7 and 8
Unmethylated CpG DNA motifs	Prokaryotic genomes and viral DNA	TLR 9

*ds* double stranded, *ss* single stranded

Contrary to the transmembrane TLRs, CLRs are classified as soluble or membrane bound. Soluble CLRs (e.g. galectins and collectins) have been reviewed extensively [9, 14]. The mostly studied membrane-associated CLRs are DC-SIGN (DC-specific ICAM3-grabbing non-integrin), Dectin-1 (dendritic cell-associated C-type lectin 1), Dectin-2 (dendritic cell-associated C-type lectin 2), MCL (macrophage C-type lectin) and MINCLE (macrophage-inducible C-type lectin) receptors [8, 9]. These receptors play a significant role in immunomodulatory responses, triggering the differentiation of T-helper cells (T_H_ cells) from naïve CD4^+^ T cells, through the assistance of an APC. CLRs not only recognize PAMPs but also the damaged-associated molecular patterns (DAMPs) and tumor-associated molecular patterns (TAMPs) from the host [15] during the processes of apoptosis and tumorigenesis, respectively. Glycans from a myriad of pathogens (parasitic, fungal, bacterial or viral) are recognized by CLRs (Table 2); the most common being mannan [16], ManLAM (mannose lipoarabinomannan) [17], mycobacterial cord factor [18], β-1,3-glucans [8] and α-1,2-mannose [8, 19]. In humans, DC-SIGN recognizes both mannan and ManLAM; Dectin-1 recognizes β-1,3-glucans and Dectin-2 recognizes α-1,2-mannose. In mice, mycobacterial cord factor is recognized by MINCLE and currently no glycans have been identified for the human MINCLE. We can observe from the literature review available, there is still more work to be done to determine ligands or PAMPs that interact with specific PRRs. Scientists must pay close attention to interspecies differences in PAMP recognition by PRRs, since we can obtain unwanted immune responses once we study them at the human level. Unwanted immunomodulatory responses can compromise the patient’s outcome from the disease.

**Table 2:** PAMPs recognized by specific CLRs

Pathogen-associated molecular pattern (PAMP)	Microorganism or classification	CLRs
Mannan	Fungi	DC-SIGN
Man-LAM	*M. tuberculosis*	DC-SIGN and Dectin 2
Le^X^	*Schistosoma mansonii* and tissue ligands	DC-SIGN
Le^Y^ + LPS	*Helicobacter pylori*	
LDNF (SP)	*Fasciola hepatica*	
β-1,3-glucans	Fungi	Dectin 1
gp120	HIV-1	DC-SIGN
α-1,2-mannose	Fungi	Dectin 2
Glucosyl and mannosyl glycolipids	*Malassezia pachydermatis* and *M. furfur*	MINCLE
Mycobacterial cord factor	*M. bovis* BCG and *M. tuberculosis*	

*Le* ^*X*^ sialyl-Lewis X tetrasaccharide, *Le* ^*Y*^ Lewis Y tetrasaccharide, *LPS* lipopolysaccharide, *LDNF* fucosylated LacdiNAc

In the vaccine development field, we can identify the importance of collaboration between medicinal chemistry, immunology and formulation science. This article will present and discuss several parameters that play prominent roles in liposomal vaccine development. Liposome size, charge and bilayer composition are some of those parameters to be discussed. Evidence will be presented to the reader for understanding of the mechanisms or effects of such liposome physicochemical characteristics, which impact vaccine development, safety, integrity and efficacy. Furthermore, we present different applications of liposomal vaccine studies that have been published for the treatment of certain infections of distinct etiological origins (viral, bacterial, fungal and parasitic). Finally, the article presents a special section on the development of subunit cationic liposomal vaccines for the prophylactic treatment of tuberculosis infections, one of the most sought-after indications in contemporary vaccinology. The section for tuberculosis vaccine development is focused on DDA-based liposomes; and additional information is available that presents other lipids or phospholipids [20, 21]. The manuscript objective is to present liposomal formulations that are not currently commercially available and inform the scientific community about liposomal formulation for early vaccine development. To date, there are only two commercially approved liposomal vaccines Epaxal and Inflexal, both by Crucell/Berna Biotech. We recommend the reader to further their knowledge in commercially available liposome-based vaccines with another review [22], which discusses the topic extensively.

## Liposome design

**Fig. 1:**
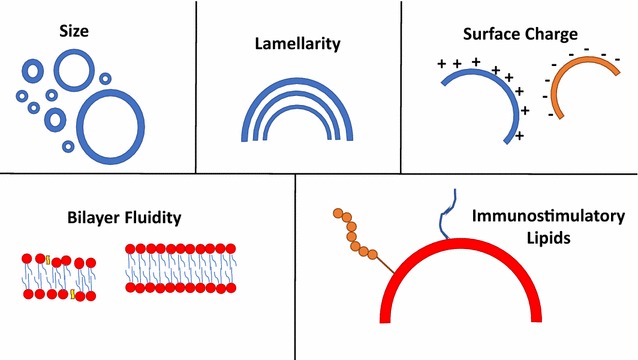
Physicochemical and morphological factors to consider in liposomal vaccine design

When designing liposomal vaccines, we should take into consideration certain factors within the liposome structure and its physicochemical properties (Fig. 1). Previous reviews on the topic have discussed the different parameters that could affect the functions and efficacy of liposomes as vaccine agents [23, 24]. Liposomal subunit vaccines are safe, with low reactogenicity, biodegradable and versatile. Reactogenicity refers to the low incidence of expected immune responses, causing symptoms like allergies, fever or pain at injection site among others. This type of vaccine contains antigen(s) (either a protein, lipid, lipopeptide etc.) from the pathogen of interest that is incorporated (depending on the antigen physicochemical nature) in the lipid bilayer or core of the liposome. The liposome will serve as an adjuvant, which potentiates the immune responses of the vaccine, improving its efficacy. Antigen incorporation can be achieved by covalent lipid conjugation (at pre- or post-vesicle formation), non-covalent surface attachment (by antibody–epitopes interactions), encapsulation, electrostatic interactions (with lipids of opposite charge) or surface adsorption. Seminal articles published earlier covered the effects of antigen encapsulation or adsorption on innate immune response differentiation [25–27]. Researchers reported that both incorporation methods dichotomously induced immune responses that enhanced T cell differentiation, when albumin was used as the incorporated model antigen in the liposomal formulations. However, when antigen size and complexity decreases (like in virus- or tumor-derived antigens), a surface adsorption incorporation method will induce better immune responses than encapsulation [28, 29].

### Liposomal vesicle size

The factors, or parameters, affecting the function and potential use of a liposome-based vaccine due to their influence in immune responses include liposome size, lamellarity, surface charge, fluidity of the bilayer, formation of lamellar-hexagonal bilayers and the addition of immunostimulatory lipids. Regarding size, it has been previously discussed that larger vesicles (> 2 µm) loaded with a tuberculosis (TB) antigen are likely to induce cell proliferation and low IL-10 induction, contrasting with vesicles around 500 nm that promoted a distinct set of cytokines (IL-1β and IFN-γ) [30]. A study by Brewer et al. presented the effects on immune response differentiation of large (> 225 nm) vs. small (< 155 nm) lipid vesicles [31]. Larger vesicles induced IL-12 cytokine production but smaller vesicles did not. Murine experiments revealed that large vesicles induced T_H_1 responses due to increased levels of IgG2a and IFN-γ. The smaller particles in this study induced a T_H_2 immune response due to increase of IL-5 and IgG1 levels. IL-1β triggers a T_H_17 immune response, whereas IFN-γ induces a T_H_1 immune response. Therefore, liposome size affects the differentiation of cellular immune responses, rendering this physical parameter a key role in liposomal formulation function.

Vesicle size could be affected by the storage period that the vaccine undergoes and by other environmental parameters. It is well recommended to perform stability studies in different environmental conditions to ensure that liposomal vaccine vesicles do not change over time [32]. Applying several methods during the formulation design and development (like spray drying, sterilization, cryoprotection and PEGylation) will lead to liposomal vesicle stabilization [33–35]. These methods or approaches mentioned earlier avoid unwanted changes in vesicle morphology that could subsequently affect the immune responses of the vaccine. Furthermore, vesicle size stability can be affected by the vesicle lipid composition [36]. An effect observed with unstable lipid vesicles is coalescence, but real-time methods have been developed to study the phenomenon and control stability [37]. Additional work has discussed vesicle stability extensively and we recommend the reader to review the appropriate literature [38–40].

### Lamellarity nature of the liposome

The lamellar nature of the liposomal vesicle could also affect the immune system causing differential responses. Beck et al. studied the adjuvanted immune responses to a recombinant HIV protein, CN54 gp140, in small unilamellar (SUV) and large multilamellar vesicles (MLV) [41]. The liposomes were composed of different combinations of monophosphoryl lipid A (MPLA) and lipids (1,2-dimyristoyl-sn-glycero-3-phosphocholine, DMPC; 1,2-dimyristoyl-sn-glycero-3-phospho-(1ʹ-rac-glycerol), DMPG; and cholesterol, Chol), with or without the addition of the saponin QS21. SUVs without QS21 could induce immune responses (characteristic of a T_H_2 cell-mediated response) to CN54 gp140 protein due to high antibody production, contrasting to MLVs. Adding the saponin QS21 restored immune responses in MLVs (higher IgG1 > IgG2a and IFN-γ titers), stimulating both T_H_1 and T_H_2 responses. The saponin did not influence SUVs immune response profile. Shek et al. presented one of the first experiments that compared vesicle lamellarity characteristics against antibody formation enhancement [42]. Liposomes composed of lecithin, dicetyl phosphate and cholesterol were prepared in the presence of bovine serum albumin (BSA). Animals injected with blank liposomes (no BSA) did not generate a significant immune response, as predicted. However, animals injected with BSA-loaded unilamellar vesicles (ULVs) generated strong immune responses compared with multilamellar vesicles (MLVs). Another seminal article presents the co-formulation or adsorption of bovine herpesvirus 1 proteins to large unilamellar (LUVs) and multilamellar (MLVs) liposomes composed of phosphatidylcholine (PC) as the main lipid component [43]. Strong antibody titers were detected in animals injected with LUVs prepared with virus proteins (both adsorbed and co-formulated) and egg PC. Recently, another study demonstrated the effects of the lamellar state for liposomes in subunit vaccines to induce immune responses [44]. SUVs with ovalbumin (OVA) induced greater levels of CD8^+^ IFN-γ responses against the protein in the spleen. Researchers added TLR3 and nine agonists, enhancing the immune responses in MLVs but not SUVs. Altogether, the studies demonstrate the effect of lamellarity of liposomes in immune responses, being SUVs the preferred state to potentiate innate and adaptive responses which improves vaccine efficacy.

### Surface charge

The surface charge of liposomes would be of important consideration for appropriate vaccine design. The overall charge can determine the adsorption or antigen interaction with the liposomes (e.g. anionic antigens will prefer to interact with cationic lipids) which affects antigen loading in the vaccine [18]. Hussain et al. found that replacing the cationic lipid DDA with the neutral lipid distearoyl-sn-glycero-3-phosphocholine (DSPC) decreases the amount of the tuberculosis recombinant antigen H56 from 84 down to 15%. In addition, the function of the vaccine in relation to immune response induction is well documented. Joseph et al. were studying an intranasal influenza vaccine model based on the liposomal formulation of the HN antigen with the polycationic sphingolipid ceramide carbamoyl-spermine (CCS) or other monocationic, neutral and anionic lipids [45]. Neutral and anionic lipid-based formulations were not immunogenic upon intranasal administration in a murine model. However, two out of five monoccationic-based liposomal formulations (containing lipids 1,2-dimyristoyl-3-trimethylammonium-propane, DMTAP; and 1,2-dioleoyl-3-trimethylammonium-propane, DOTAP) induced vigorous local and systemic immune responses (T_H_1 and T_H_2 type responses). Researchers compared the monocationic liposomal formulations with the CCS-based liposomal formulation and the only commercially available influenza vaccine, demonstrating the efficacy of the sphingolipid as an immunopotentiator with higher antibody titers and protective immunity for approximately 9 months. Another study with Newcastle disease virus compared the immunization effects in chickens of neutrally- (EPC-Lip), anionically- (PS-Lip) and cationically-charged (SA-Lip) liposomes [46]. Strong humoral responses (local and systemic) were observed in neutral formulations of EPC-Lip, contrasting to the cationic SA-Lip. The anionic formulation mainly composed of phosphatidyl serine (PS-Lip) elicited the higher hemagglutination titers. Recently, Hussain et al. determined that replacing the cationic lipid DDA with the neutral DSPC reduced the T_H_1-mediated immune response of the formulation [18]. Based on the studies presented above we can conclude that cationic formulations might be the most suitable option to elicit strong immune responses, increasing antibody titers. There is additional information available about cationic lipids, like DDA, which possess significant immunostimulatory and adjuvanting properties [47, 48] and further explanations will be provided ahead.

### Bilayer fluidity

The bilayer fluidity, dependent on the lipid gel-liquid crystal transition temperature and its effects on immune responses, is of important interest when designing efficacious vaccines. Many published works have evaluated and described this phenomena, including the earliest work by Yasuda et al. [49]. The effects on immune responses of liposomes prepared with phospholipids of PC with different transition temperatures containing the hapten Dnp-Cap-PE were measured. DMPC, DPPC (1,2-dipalmitoyl-sn-glycero-3-phosphocholine) and DSPC (all with high transition temperatures T_m_ > 20 °C) were favorable in eliciting antibodies to the hapten, contrasting to results obtained with DOPC (1,2-dioleoyl-sn-glycero-3-phosphocholine), DLPC (1,2-dilauroyl-sn-glycero-3-phosphocholine) and EPC (T_m_ < 0 °C). Another research team followed a similar experimental design preparing liposomes with low- (DOPC and DLPC, − 20–0 °C), intermediate- (DPPC and sphingomyelin, 25–40 °C) and high-transition temperature (DSPC, > 50 °C) lipids [50]. DMPC, DPPC and sphingomyelin induced immune responses as per a plaque-forming assay, but DSPC was a poor immunogen. Cholesterol was added to the liposomal formulations, inducing significant humoral immune responses. The results from these two groups produced different outcomes: Yasuda et al. [49] presenting that less fluid lipids are better immunogens than fluid phospholipids and van Houte et al. [50] establishing that intermediately fluid phospholipids (which have a phase transition temperature of 25–40 °C) are better immunostimulatory agents. This discrepancy might be attributed to other factors, like particle size and Zeta potential, but such factors were not reported or analyzed to determine their role in these studies.

Additionally, Mazumdar et al. revealed that liposome composition may have an effect on immune responses [51]. Here, researches incorporated a leishmanial antigen (LAg) in liposomes containing DMPC, DPPC or DSPC and described the immunization process and results in a hamster model. No significant delayed hypersensitivity was detected in DMPC- or DPPC-containing lipids, which contrasted with DSPC-containing lipids. Moreover, DSPC-containing lipids protected up to 95% of the hamsters against a leishmanial infection. Recently, Kaur et al. presented results on how cholesterol influences the bilayer fluidity [52]. For instance, a direct correlation of cholesterol and membrane fluidity was observed in DDA:TDB (trehalose dibehenate) liposomal formulations. However, less IgG was detected as cholesterol increased in the system after 12 days of immunization in mice. This effect might be due to the loss of antigen in more fluid (high cholesterol) liposomes as the authors pointed out. The cytokine IFN-γ was at elevated levels when cholesterol was not present in the lipid bilayer. Even with the compelling evidence of how transition temperature and lipid bilayer composition affects immune responses, we can find conflicting results in published data. For example, Hampl et al. found no significant influence of immune response induction based on liposomes containing phospholipids with different transition temperatures [53]. Although the data presented does not follow a clear pattern, the general conclusion is that the more fluid a liposome is the less immune response it will generate when administered in different animal models. Likely behind this phenomenon is that fluidity tends to increase the release of the antigen from the liposomes, affecting antigen presentation and consequently the strength of immune responses. We need to point out that cholesterol also participates as a bilayer stabilizer, increasing rigidity in biological membranes [54]. Therefore, we might consider that the increase in bilayer fluidity by cholesterol observed in the studies discussed above may occur in synthetic bilayers only. Further research must account for such correlation due to the complexities of biological membranes.

### Immunostimulatory lipids and liposome deposition

Immunostimulatory lipids might be of value in the vaccine design process. The lipids can act as adjuvants in the vaccine formulation and enhance the immune response we are looking for (innate or adaptive) as previously reported and reviewed [2, 5, 6, 47]. In a study developed by Rao et al., two adjuvants (lipid A and CpG-containing oligodeoxynucleotides, CpG-ODN) were studied in a liposomal formulation based on the HIV envelop protein ogp140 [55]. Both lipid A and CpG-ODN-containing liposomes elicited six and threefold anti-ogp140 antibodies, respectively. Immunization of BALBc mice with the HIV antigen incorporated in lipid A-containing liposomes produced a mixed T_H_1/T_H_2 immune response. Combining both adjuvants in the liposomal formulation generated a T_H_1 immune response. Puangpetch et al. presented the effect of using zwitterionic or cationic lipids in vaccine liposomal formulations [2]. Researchers compared DOTAP (cationic phospholipid) vs. DOPC (neutral phospholid) containing the adjuvant CpG-ODN. DOTAP-based liposomes with adjuvant enhanced the immune response against *Burkholderia pseudomallei*, which suggest an alternative approach for the treatment of melioidosis. The published data available suggests that certain lipids can induce, or enhance, immune responses. Very important is the fact that cationic liposomes are the ideal model to design effective vaccines due to their immunostimulatory properties.

Finally, the immunostimulation of the vaccine can be affected by the deposition of liposomes at the site of injection which in turn is affected by particle size. Henriksen-Lacey et al. reported this in two separate articles [6, 56]. On Henriksen-Lacey et al. researchers utilized the phospholipid DDA as the building block for the liposomes containing the adjuvant TDB. The antigen for tuberculosis infection, Ag85B-ESAT-6, was co-administered or incorporated to liposomes in mice. Antigen administered alone to mice did not created a depot at the site of injection, causing the antigen to diffuse away from the site and reduce immune responses. The observations directed them to conclude that cationic liposomes made up of DDA promotes depot formation at the site of injection mainly due to size characteristics between antigen-loaded liposomes and antigen administered alone. Later, Henriksen-Lacey et al. [30] studied the effect of liposome composition [DOTAP, DDA or DC-Chol (dimethylaminoethane-carbamoyl-cholesterol)] and the depot formation and antigen distribution. DDA and DC-Chol represented the phospholipids where liposomes induced the migration of monocytes to the site of injection and a significant increase of IFN-γ levels. In general, larger liposomes will form a depot at the site of injection meanwhile smaller liposomes will migrate to lymphoid tissue for antigen presentation and processing.

## Liposome-immune cell interactions to improve vaccine effectiveness

**Fig. 2:**
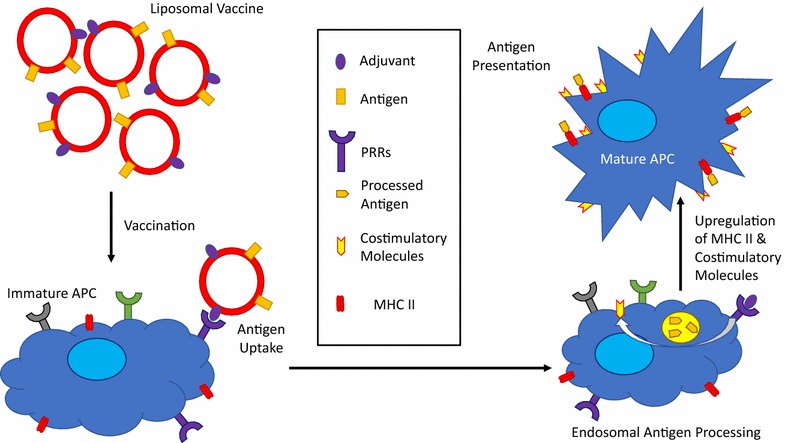
Co-delivery of antigen and adjuvant to APCs in subunit vaccines. Interaction of the adjuvant with the PRR results in upregulation of co-stimulatory molecules necessary for appropriate T cell stimulation

Early vaccination strategies included the development of attenuated or inactivated vaccines, which are composed mainly of weakened or dead pathogens, respectively. Currently these kinds of vaccines are facing challenges with variabilities in immune response induction [57, 58]. Modern vaccination strategies are emphasizing the study of subunit vaccines and proving their effectiveness in immune response modulation [59, 60]. Subunit vaccines are characterized by the co-delivery of adjuvants and antigens for immunostimulatory purposes (Fig. 2). The antigen is either a natural or recombinant peptide, protein or molecule derived from the pathogen. The adjuvant will enhance the immune response of the vaccine and potentiate the effect of the antigen during the process. It is very important to know how to present the antigen incorporated in the liposomes to APCs because this will ensure their proper immune cell maturation, antigen presentation and eventual induction of adaptive immune responses as previously reviewed [61, 62]. Cell targeting studies have been investigated and produced significant data and observations of how antigen presentation plays a significant role. Aramaki et al. investigated the utilization of liposomes as carriers for antigens related to gut-associated lymphoid tissue and their uptake by rat Peyer’s patches [63]. Preferential uptake was observed for liposomes in the rat Peyer’s patches than non-patch tissues, specifically for DSPC:PS:Chol liposomes. This suggests that liposome composition affects their uptake. Fluorescently labeled liposomes were internalized by patch tissue in the lower ileum with size (> 374 nm) playing an important direct correlation with uptake. Another report looked at how cationic vesicles composed of DDA could interact with normal and transformed mouse fibroblasts cells [64]. Cell–cell adhesion was observed when DDA concentration was equal or greater to 50 µM. Investigators determined that normal cells were susceptible for DDA vesicles meanwhile transformed cells were resistant to DDA-mediated (> 1 mM) cell death. The interaction with cationic vesicles created a change in cell charge from anionic to cationic, making this system of cationic vesicles ideal for the delivery of negatively charged macromolecules like proteins and DNA.

### Vaccine interaction with neutrophils, monocytes and APCs

Several studies presented results on how liposome-based vaccines interact with neutrophils and monocytes, key players in inflammation and immune responses. First, Karathanasis et al. used previously characterized and purified peptides to target liposomal nanocarriers to some types of leukocyte cells [65]. Peptides were covalently attached through the carboxyl group of DSPE-PEG by utilizing the crosslinker *N,N*-dicyclohexylcarbodimide (DCC). Researchers found that targeted liposomes interacted better with monocytes and neutrophils, contrasting with results obtained by non-targeted liposomes. Moreover, the surface density of the peptide directly correlated with liposomes-cell interactions, making this parameter a new way to measure the effectiveness of the interactions and the potential induction of immune responses valuable in subunit vaccine development. Then, Johansen et al. investigated the targeting of monocytes and the delivery of a TLR agonist (TMX-202) using a cationic liposomes-based formulations (mainly POPC (1-palmitoyl-2-oleoyl-sn-glycero-3-phosphocholine):DOTAP composition) [66]. After an hour incubation, the subset of monocytes targeted by the liposomes were lymphocytes and granulocytes (75–95%). A strong IL-6 and IL-12p40 induction was observed, accompanied by monocyte differentiation to CD14^+^/DC-SIGN^+^ DCs. Mainly found in the lymph nodes, lymphocytes include natural killer (NK), T and B cells, which are involved in innate immune responses, cell- and humoral-mediated immunities, respectively. Granulocytes, or polymorphonuclear (PMNs) leukocytes include basophils, neutrophils, mast cells and eosinophils that participate in allergic and inflammatory reactions. The information gathered by the researchers is important because it can determine future treatments and vaccine developments to focus on selected immune responses, depending on the cell or cell types that are being targeted, reducing or avoiding unwanted adverse reactions (e.g. allergies or chronic inflammation).

After interaction with the antigen, cytokines and/or interaction with their milieu, monocytes could differentiate into macrophages or dendritic cells. Both cell types will then migrate to lymph nodes to elicit the corresponding adaptive immune responses. Macrophages and dendritic cells are specialized APCs that reside in the blood stream and help in antigen uptake and antigen presentation to T and B cells, inducing cell-mediated and humoral immune responses, respectively. It is known that antigen-containing liposomes are internalized by pinocytosis in macrophages and then cross presented to CD8^+^ T cells (specialized T cells that attack and kill tumors), inducing antigen-specific cytotoxic T lymphocytes [67]. This cross-presentation occurs when exogenous antigen is up taken and presented via the class I major histocompatibility complex (MHC I), which classically only happens with endogenous antigens such as those from viruses. To cross-present and elicit a CD8^+^ T lymphocyte response, the antigen must be delivered to the cytosol APCs. This was studied by Owais et al. in which yeast-derived lipids liposomes and egg PC:Chol liposomes were tested against J774 A1 macrophages and the interactions measured [68]. The fusion rate for yeast lipid liposomes to macrophages was at 40–70%, compared to 1–8% for egg PC:Chol liposomes. Liposome contents were successfully delivered to the cytosol of macrophages. Ovalbumin was used as the model antigen for yeast-derived lipid liposomes and it was found that it elicited a strong CD8^+^ T cell response. Another group of researchers investigated the uptake mechanisms of liposomes in rat peritoneal macrophages (PM) [69]. Researchers determined that two uptake systems exist because of cholesterol content and size differences of liposomes. For high- (44% molar) and medium-cholesterol (33% molar) content liposomes, the complement receptor-mediated phagocytosis occurs. A complement-independent uptake pathway was suggested for low-cholesterol content liposomes since no inhibition of their internalization rate was observed by the anti-C3 antibody. Therefore, once again, lipid composition is a main player in liposome-cell interactions, potentially affecting the way immune responses develop.

### Mannosylated liposomes

Mannosylated liposomes are becoming an alternative method to deliver antigens or antimicrobials to macrophages or DCs [70, 71]. This approach is due to the fact that mannose receptors can be found in these cell types, improving directed-targeting [16]. Other research teams have used cationic lipids like DDA, DOTAP and/or DC-Chol to enhance or potentiate the immune responses due to cell targeting strategies [48, 72, 73]. Korsholm et al. determine that DDA liposomes were minimally internalized by T cells in the mixed splenocyte cultures, contrasting to high uptake rates for APCs (bone marrow dendritic cells) through class II MHC (MHC II), leading to an enhanced OVA presentation. The immune responses described by Varypataki et al. contrasted to Korsholm et al. since DOTAP:DOPC liposomes bearing the peptide SIINFEKL and polyI:C assisted in the delivery of OVA to DCs through MHC I, inducing a CD8^+^ T cell response.

Understanding and applying innovative ways of cell targeting could improve the discovery pipeline for novel therapeutic agents, avoiding undesirable immune responses that can be detrimental to the patient. These therapies could treat inflammatory diseases [like arthritis or chronic obstructive pulmonary disease (COPD)], infections or cancer. The following sections will focus on how liposome-based vaccines are being utilized for the treatment of infections from viral, bacterial, fungal and parasitic origins. A special section will discuss liposomal vaccines that target the bacterium *Mycobacterium tuberculosis* to treat TB infections.

## Viral infections and liposomes-based vaccines

Viruses are ethiologic agents of different diseases in animals [74] (including humans [75, 76]), plants [77], parasites [78, 79] and bacteria [80, 81]. The basic definition of a virus is a non-living infectious agent that requires living cells for its replication and survival, which allows spreading of the disease. This cell lysis leads to inflammation and tissue damage which are detrimental for the host, enhancing the state of the disease. Additionally, some viruses, like HIV, destroy immune cells hindering effective immune responses when other infections (secondary infections in seropositive patients) occur. Viral infections can be treated by either antiviral therapeutic [82] or by prophylactic vaccine [83] approaches. Here, we will present several examples of vaccine applications based on liposomes for the prophylactic treatment of certain viral infections.

### Hepatitis

One of the most significant viruses that affect human health are hepatitis viruses. Hepatitis is an inflammation of the liver tissue caused by the five types of hepatitis viruses (A, B, C, D and E). Hepatitis A and E are spread through contaminated food or water sources. Hepatitis B (HBV) is sexually transmitted or during pregnancy and birth. Both HBV and hepatitis C can be transmitted through blood (needle exchange by IV users) and hepatitis D can only infect people infected with HBV. Two seminal reports investigated novel approaches for the prophylactic treatment of HBV utilizing cationic lipids. Brunel et al. reported the effectiveness of recombinant hepatitis B surface antigen (HBsAg) presentation based on DC-Cholesterol liposomes or aluminum hydroxide (alum) adjuvants in a subcutaneous vaccine model [84]. The DC-Chol-based vaccine elicited antibody (IgG1 and IgG2a) titers in three mice lines (BALB/c, OF1 and B10.M). Compared to the alum-based vaccine, which demonstrated weak immunogenicity, DC-Chol liposomes induced controlled T_H_1 and T_H_2 immune responses characterized by normal, but significant levels of cytokines IL-2 and IFN-γ and IL-5, respectively. Controlled immune responses are important to avoid inflammation that could result in tissue damage. The researchers concluded that cationic lipids like DC-Chol could be used as adjuvants, enhancing the immunogenicity of previously non-immunogenic vaccines, specifically in the development of prophylactic vaccines against hepatitis B virus. Another group investigated the use of a transcutaneous vaccine for the treatment of HBV infections [85]. In that report, the vaccine presents some differences from the Brunel et al. article based on the antigen and carrier types. First, cationic transfersomes, a type of liposome, were prepared from DOTMA (1,2-di-*O*-octadecenyl-3-trimethylammonium propane) phospholipid and sodium deoxycholate (SDC) at different DOTMA weight ratios (75–95% w/w). Second, plasmid DNA encoding the HBsAg gene was loaded to the transfersomes instead of the antigen. The transfersomes were not cytotoxic to HepG2 cells and were stable at different temperatures (4 and 28 °C). Immunization studies included HBsAg DNA-loaded tranfersomes (topical), naked HBsAg DNA (topical and intramuscular) and pure HBsAg (intramuscular) administered to BALB/c mice. Significant levels of anti-HBsAg antibodies and cytokines (IL-2 and IFN-γ) were elicited in topical DNA-loaded transfersomes as compared to intramuscular naked DNA delivery. This antibody and cytokine profile confirmed the induction of T_H_1 and T_H_2 immune responses as observed in Brunel et al. Both studies represent great advances in the treatment of HBV infections with novel approaches and administration routes that could applied in the future as preventive vaccines.

### Influenza

Influenza is an infectious disease caused by the influenza virus, affecting human health with a pandemic effect in several instances. The virus is divided in three types (A, B and C) and it is spread through the air from coughs and sneezes, therefore the importance for vaccine development studies for the infection. An intranasal (i.n.) liposomal influenza vaccine study was presented by Joseph et al. [45]. The vaccine formulation developed by the group was based in the polycationic sphingolipid *N*-palmitoyl-d-erythro-sphingosyl-carbamoyl-spermine (or ceramide carbamoyl-spermine, CCS) and was compared with other formulations containing monocationinc phospholipids (DC-Chol, DDA, DSTAP (1,2-stearoyl-3-trimethylammonium-propane), DMTAP and DOTAP). Cholesterol was added to increase liposome fluidity and the lipids DMPC and DMPG were included in neutral and anionic formulations, respectively. All formulations contained the influenza A antigens hemagglutinin and neuraminidase (HN). DMTAP- and DOTAP-based vaccines were the only monocationic lipid formulations to induce strong systemic (serum) and local (lung) T_H_1 and T_H_2 responses. Surprisingly, DDA-containing formulations did not induce strong, or significant, local or systemic immune responses. No specific reasons were provided by the team for such results but we can infer that vesicle morphology (multilamellar and oligolamellar cationic formulations), size (1–4 µm diameter) and encapsulation efficiencies (10–90% for non-CCS cationic formulations) would be responsible. The CCS-based vaccine formulation was the only formulation to be at the same or superior level of effectiveness compared to the commercially available vaccine with cholera toxin as the adjuvant. More recently, researchers investigated a triple co-culture model of the human respiratory tract to study the immunostimulatory responses of virosomes and liposomes and their internalization [86]. The epithelial cell line 16HBE was grown with monocyte-derived macrophages (MDMs) and dendritic cells (MDDCs) and exposed to liposomes and virosomes to evaluate the immune responses elicited by the nanocarriers. The virosomes were liposomes prepared with solubilized influenza A/Brisbane/59/2007 H1N1 membrane proteins and included the neutral lipids DOPC and OPPE (1-palmitoyl-2-oleoyl-sn-glycero-3-phosphoethanolamine, POPE). Liposomes were prepared with previously mentioned neutral lipids and no influenza soluble membrane proteins were added. Virosomes were internalized more efficiently by all cell types in mono- and co-cultures, with APCs like MDMs and MDDCs presenting the highest internalization levels as per flow cytometry and laser scanning microscopy. MDDCs were moderately activated by liposomes and virosomes in monocultures, and inducing elevated levels of cytokine (IL-1β and IL-8) production in co-cultures. Virosomes were internalized at higher levels in epithelial cells in comparison to liposomes.

### Respiratory syncytial virus

Another respiratory viral pathogen of interest is the respiratory syncytial virus (RSV). RSV causes respiratory tract infections, specifically lower respiratory tract infections in children and it presents high hospitalization incidence [87]. An i. n. study was developed by Klinguer et al. in which cationic DDA liposomes were mixed with the recombinant fragment of the RSV G protein (BBG2Na) and administered to BALB/c mice [88]. The DDA + BBG2Na liposomal formulation presented significant antibody (IgG and IgA) titers at systemic (serum) and local (nasal) levels. Cytokine production was higher for IL-2 and IFN-γ in cationic liposomes carrying the RSV recombinant antigen, confirming the protection after a viral challenge and the induction of T_H_1 immune response. These three reports on respiratory viruses and their prophylactic vaccine development confirms the importance of mucosal immunization as it elicits local and systemic B- and T-cell responses [89]. It is also clear that cationic phospholipids like DDA, DC-Chol or DOTAP play a leading role (Table 3) in the immunostimulation of subunit vaccines and more approaches should be investigated. Different administration routes were investigated in the studies for viral infections. However, we cannot verify and compared these administration routes to determine the best alternative due to experimental differences in the studies (liposomal composition and targeted virus). Future experiments should focus their efforts in comparing different administration routes with the same liposomal composition and prophylactic treatment of a particular virus.

**Table 3:** Promising vaccine formulations for the treatment of viral infections

Lipid(s) and sterol used	Virus type	Cell line/animal model used	Administration route	Promising liposome formulation	References
DOTMA	Hepatitis C	HepG2 cells and BALB/c mice	Transcutaneous	DOTMA	[85]
DC-Chol	Hepatitis B	Lymph node cells, BALB/c, OF1 and B10.M mice	Subcutaneous	DC-Chol	[84]
DMPC, DMPC/DMPG, DC-Chol/DOPE, DSTAP/Chol, DDA/Chol, DOTAP/Chol, DMTAP/Chol and CCS/Chol	Influenza H3N2	Splenocytes from BALB/c and C57BL/6 mice	Intranasal	DMTAP/Chol and DOTAP/Chol	[45]
DDA	Respiratory Syncytial Virus	BALB/c mice	Intranasal	DDA	[88]
DOPC/OPPE (Influenza virosome) and DOPC/OPPE (liposomes)	Influenza	MDMs, MDDCs, 16HBE14o cells, PHNECs and EPCam + cells	N/A	DOPC/OPPE (Influenza virosome)	[86]

Cell cultures and no vaccination in animal models were employed

*N/A* not applicable

## Bacterial infections and liposomal vaccines

Bacteria can be beneficial for humans, but also detrimental to our health when they harbor pathogenicity traits. Bacteria are divided in Gram positive (+) or negative (−) based on Gram staining, that surveys the peptidoglycan content in the cell wall. Gram (+) bacteria have a positive result in the Gram Stain method, which determines in a qualitative way the presence of the cell wall component: a thick peptidoglycan layer. In contrast, Gram (−) bacteria present a negative result in the stain, indicating a thin peptidoglycan layer, sandwiched between two cell membranes (plasma and outer membranes). Gram (−) bacteria also contain an important immunogenic molecule and pathogenicity factor, the lipopolysaccharide (LPS) [90, 91]. LPS has been used to increase the fusogenicity of cationic liposomes [92]. In that study, researchers developed a carrier system to incorporate LPS into mammalian cell membranes via DOPE (1,2-dioleoyl-sn-glycero-3-phosphoethanolamine):DOTAP (1:1 wt. ratio) liposomes. The team of researchers demonstrated that high LPS concentrations on immortalized fibroblasts generated the activation of macrophages, starting the elimination of LPS-bearing cells.

Additionally, bacteria will have unique cell components, like genetic material, lipids or proteins, that could serve as adjuvants or antigen markers for subunit vaccine development, depending on the molecule chemical characteristics. Li et al. investigated the potential use of cationic (DDA-based) mannosylated liposomes to deliver the model DNA plasmid pGL4.10 (encoding *luc2*) [19]. The plasmid was protected from nuclease degradation by the liposomes. The cationic mannosylated liposomes showed high uptake and transfection, activating bone marrow DCs (BMDCs). BMDCs activation was characterized by the upregulation of CD80, CD86 and CD40. Another study by Nakanishi et al. studied the effects of positively, negatively and neutrally charged liposomes administered subcutaneously in the immune responses of the fragment A of diphtheria toxin (DTA) and ovalbumin (OVA) [93]. Cationic liposomes were composed of phosphatidylcholine:cholesterol:stearylamine (PC:Chol:SA, 4:5:1 molar ratio), anionic liposomes were composed of PC:Chol:l-α-dimirystoylphosphatidic acid (PC:Chol:DMPA, 4:5:1) and neutral liposomes were composed of PC:Chol (1:1). Positively charged liposomes could induce potent antigen-specific cytotoxic T cell responses. However, DTA-containing cationic liposomes were cytotoxic to macrophages. In contrast, empty cationic liposomes or DTA-loaded anionic and neutral liposomes were not cytotoxic. CD8^+^ OVA responses were highly induced by positively charged liposomal vaccines, potentially presenting the processed antigen through MHC I. Both research articles presented us the utilization of liposomes to investigate and test the effects on immune responses during vaccination. The immune responses measured varied, but making it clear that cationic liposomes induced the required cells (macrophages and DCs) to obtain the appropriate responses.

The liposomal vaccine studies mentioned above serve as the basis for the following articles which incorporate bacterial antigens for the development of prophylactic vaccines for certain infections. Puangpetch et al. developed a cationic-based liposomal formulations incorporating CpG ODN and to determine the prolongation and mechanisms of the immune responses [2]. The researchers employed the etiologic agent of melioidosis, *Burkholderia pseudomalei*, as the infection model in BALB/c mice. Cationic and not neutral liposomes administered intramuscularly granted protection against the bacterial challenge study. Prominent levels of IFN-γ were observed 2 days postinfection, but lowered by a CpG ODN-loaded cationic liposome pre-treatment. Neutrophils were not activated by the cationic liposomes with CpG ODN, but macrophages were stimulated by the formulation due to nitric acid production and low intracellular bacterial burden (30 days post vaccination). An additional study involving mannosylated liposomes containing the meningococcal PorA (from *Neisseria meningitidis*) focused its attention on cell interaction [94]. Anionic (PG- (phosphatidylglycerol) and PS-based) and cationic (DMTAP-based) liposomes were formulated, and one of the anionic formulations was mannosylated (PC:PG:Chol + Man-PE (mannosyl phosphatidylethanolammine). When exposing the formulations to human and murine DCs, researchers observed an increase of liposome-cell interaction in the anionic mannosylated liposomes and cationic liposomes when compared to anionic formulations alone. The result indicated that adding mannosyl moieties to liposomes generated a mannose receptor (MR)-mediated cell interaction. The murine DCs were confirmed to present the markers MHC II^+^, CD11c^+^ and CD11b^+^, meanwhile human DCs presented CD40^+^, CD1a^+^ and both MHC I and II. Researchers in the field should address studies that investigate route of administration effects on immune responses. With the studies presented here, it is difficult to determine what best route of administration we should follow for future prophylactic vaccine development. We recommend investigating the optimized formulations in different administration route studies.

The use of cationic liposomes seems of importance for the development of adequate vaccine formulations. The studies presented above demonstrate that by employing cationic phospholipids like DOTAP, DMTAP and DDA, would improve cell interaction levels, allow adequate antigen presentation and induce strong immune responses. These effects will insure that the vaccine will work properly and optimally. For the benefit of the reader, Table 4 presents a summary of the most relevant literature that optimized vaccine formulations for the treatment of bacterial infections. Additionally, previous research on cationic liposomes have discussed the cytotoxicity potential of such formulations. DDA has been determined to be safe as no relevant cytotoxic effects were determined in studies by Hilgers and Snippe and Gall [47, 95]. Only local inflammatory reactions manifested as swelling were observed in mice (when administered alone). Contrasting results are found for DOTAP and DOTMA cationic lipids. DOTAP has been found to be not cytotoxic to macrophages in a study by Jin et al. [96], but Romøren et al. determined that macrophage-derived cell lines were found to be affected by the cationic lipid [97]. The cytotoxic response differences might be due to structural and morphological characteristics in the formulations. Jin et al. employed Tween 20 and tricaprin (part of the solid core) to form solid lipid nanoparticles, meanwhile Romøren et al. just prepared liposomes. Further contradictory information is available for DOTMA, which can be cytotoxic for RAW 264.7 cells at all lipid ratios (DOTMA + DOPE), but not to human umbilical endothelial cells or mouse fibroblasts cells [98]. Kurosaki et al. determined that erythrocytes undergo agglutination and hemolysis when exposed to DOTMA-based liposomes [99]. However, an earlier study by Kurosaki et al. determined lower cytotoxicity for erythrocytes, showing no agglutination and hemolysis, when DOTMA was formulated with *N*-laurylsarcosine, and Chol, vitamin E and Chol or egg PC and Chol [100]. Future work should include the analysis of lipids alone and formulated with other lipids to elucidate the cytotoxic effects. Additional work should be done for these and other bacterial infections lacking proper prophylactic vaccines, leading to outcome improvement from the infection.

**Table 4:** Promising formulations for the treatment of bacterial infections

Lipid(s) and sterol used	Bacteria or disease	Cell line/animal model used	Administration route	Promising liposome formulation	References
PC/PG/Chol, PC/PG/Chol/Man-PE, PC/PS/Chol, PC/DMTAP/Chol	Meningitis	Monocyte-derived human DCs and murine bone marrow-derived DCs	N/A	PC/PG/Chol/Man-PE and PC/DMTAP/Chol	[94]
PC/Chol/SA, PC/Chol/PA and PC/Chol	Diphteria toxin	BALB/c, C57BL/6 and ddY mice; P815, P13.1 and CD8OVA cells	Subcutaneous	PC/Chol/SA	[93]
DOTAP and DOPC	Melioidosis	Neutrophils and splenocytes from BALB/c mice	Intramuscular	DOTAP	[2]
DOPE/DOTAP	*E. coli*	Mouse embryonic fibroblasts (MEF) and RAW 264.7 macrophages	N/A	DOPE/DOTAP	[92]
DDA/Chol/Man-C6-Chol and DDA/Chol	*E. coli*	DC 2.4 cells	N/A	DDA/Chol/Man-C6-Chol	[19]

Cell cultures and no vaccination in animal models were employed

*N/A* not applicable

## Liposomal-based vaccines in fungal infection treatment

Fungi are eukaryotic organisms that include yeasts, molds and mushrooms. Several yeasts and molds are common pathogenic agents, especially in immunocompromised patients [101, 102]. Antibiotic resistance is being detected, not only in isolates from the USA but also in other developed countries, in different fungal species (like *Candida* and *Aspergillus*) [103]. Due to the threat of antifungal resistance, other therapeutic approaches should be investigated, and liposomal vaccines may play a significant role. To the best of our knowledge, the literature review presents liposome-based vaccines for *Candida* sp. infections but limited information is available for other fungal pathogens. Studies investigating other fungal species with elevated infection prevalence, like *Aspergillus*, *Fusarium*, *Coccidioidomyces* and *Zygomycetes* sp., have been identified but their treatment approach does not include liposomal vaccines, and we invite further reading on the topic [101, 104, 105]. We will discuss ahead information available for liposomal vaccine development for the prophylactic treatment of *Candida* sp. infections (Table 5).

**Table 5:** Candidiasis treatment with potential liposomal vaccines

Lipid(s) and sterol used	Fungi or disease	Cell line/animal model used	Administration route	Promising liposome formulation	References
PC/Chol	*C. albicans* and *C. tropicalis*	BALB/cByJ mice	Intravenous	PC/Chol	[106]
PC/Chol		BALB/c mice	Intravenous	PC/Chol	[107]
DMPC/DMPG	*C. albicans*	ICR mice	Subcutaneous	DMPC/DMPG	[108]
EPD/DOGS-NTA-Ni		BALB/c mice	Intradermal	EPD/DOGS-NTA-Ni	[109]
		ICR mice	Intradermal		[110]
DDA:MO		Macrophages and BALB/c mice	Subcutaneous	DDA:MO	[111]
		BALB/c mice	Subcutaneous		[112]

The first report dealing with the study and development of a vaccine to treat candidiasis, encapsulated the mannan adhesin portion of *Candida albicans* [106]. The adhesin protion of the mannan of two *C. albicans* serotypes (A and B) were incorporated in PC:Chol liposomes (3.2:1 molar ratio). Mice were vaccinated (intravenously) during a period of 5–6 weeks and challenged with *C. albicans* infection, presenting increasing resistance to disseminated disease. Furthermore, antiserum agglutinins (IgM-type antibodies) from the immunized mice were studied for their humoral protective characteristics against *C. tropicalis* infection, demonstrating the efficacy of the vaccine. Subsequently, researchers decided to investigate the effectiveness of the monoclonal antibodies obtained from the previous study in a vaginal candidiasis model [107]. Similarly, the vaccine (L-mann) was prepared by the mannan adhesin fraction incorporation into PC:Chol liposomes. Mice were immunized intravenously once a week for 5 weeks prior vaginal inoculation. Vaccinated mice challenged with *C. albicans* presented lower CFUs (110 ± 38 × 10^3^ CFU/g of vaginal tissue) when compared to non-mannan vaccine approach (240 ± 44 × 10^3^ CFU/g of vaginal tissue). Both article demonstrated the capacity of the vaccine to protect at local or systemic infections of *C. albicans*.

Further studies investigated zwitterionic and cationic liposomes utilizing ribosomes and recombinant Hsp90 protein as antigens from *C. albicans* [108–110]. Eckstein et al. prepared anionic DMPC:DMPG liposomes co-lyophilized with RNA obtained from cell lysates of *C. albicans* cultures or by lipid film formation. Mice protection in a subcutaneous vaccination (60% survival) against the fungal challenge demonstrated the effectiveness of the vaccine prepared by the co-lyophilization method in the presence of *C. albicans* ribosomes. Subsequently, neutrally charged metalloliposomes with incorporated recombinant Hsp90 (heat shock) protein from *C. albicans* were developed. Nickel-chelating liposomes were prepared with EPC and 1,2-dioleoyl-sn-glycero-3-[(*N*-(5-amino-1-carboxypentyl)iminodiacetic acid)succinyl] (nickel salt) (DOGS-NTS-Ni) at molar ratios of 95:5. A non-pyrogenic MDP was used as an adjuvant in the liposomal system and the protein was surface attached by metallochelating bonds. DCs interacted with the liposomes and phagocytosed the nanoparticles in vitro. T_H_1 and T_H_2 immune responses were induced after intradermal mice vaccination in comparable levels to the Freund’s complete adjuvant vaccine. Following the previous report, Knotigová et al. employed nickel-chelating liposomes with different MDP-derivatives (norAbuMDP/GMDPs) and tested their adjuvant vaccine potential [110]. Also, Hsp90 from *C. albicans* was employed as the model antigen. Adaptive and innate immune responses were induced by the developed vaccine systems in intradermal vaccinated rabbits and mice.

Recently, cationic liposomes studies incorporated cell wall surface proteins (CWSPs) of *C. albicans* and their immunostimulatory properties analyzed during subcutaneous administration [111, 112]. Both studies employed DDA:monooleoylglycerol (MO) at 33:67 molar ratio. Liposomes were not toxic to macrophages and were internalized within 20 min of exposure. In the first study, immunized mice displayed strong humoral- and cell-mediated immune responses. Antibodies were produced against cell wall proteins Cht3p and Xog1p. In the second report, two CWSP-loaded cationic liposomal formulation (ADS1 and ADS2) were tested against disseminated candidiasis. ADS1 immunized mice presented significantly higher levels of *C. albicans* antibodies, contrasting with the ADS2 formulation. This antibody titer production induced the phagocytosis of the fungus. Elevated levels of the cytokines IL-4, IL-17 and IL-10 were significantly higher than control groups, suggesting T_H_2, T_H_17 and anti-inflammatory immune responses, respectively.

Future studies on fungal infections and their prophylactic treatment with vaccines should be performed not only in *C. albicans*, but also other ethiological agents (e.g. *Aspergillus*). The literature review revealed a field with potential for growth and development of novel approaches to treat fungal infections, which benefit immunocompromised patients (elderly or HIV-seropositive patients). Additionally, further studies must employ further cationic lipids and their effects on immune response induction. Studies comparing physicochemical properties of liposomes to treat fungal infections must take place to optimize the vaccine strategy and avoid unwanted responses and results. In the studies presented above, the intravenous administration was investigated and revealed affirmative results (~ 60% survival of mice). However, we recommend further studies that compare immunomodulatory responses against mucosal vaccine administration for vaginal candidiasis infections. Similar survival rates were observed for subcutaneous and intradermal administration routes, but future studies should investigate a particular set of liposomes against the different administration to observe if survival rates are affected.

## Parasitic infections and liposomal vaccines

### Parasitism and malaria

Parasitism is a non-mutual, biological interaction in which the parasite lives inside the host and derives its own nutrients at the host’s expense. Parasites can be classified as macroparasites (visible with the naked eye) like helminths, or microparasites (which are smaller) like viruses, bacteria or protozoa. A textbook example of a parasite is *Plasmodium vinckei*, causative agent of malaria. In malaria, the parasite (*Plasmodium* sp.) is transmitted by mosquito bites. The parasite’s sporozoites reside in the liver (in humans), developing into merozoites that infect human red blood cells, initiating the red blood cell cycle. When appropriate, the merozoites will developed into gametocytes that infect more red blood cells that are taken up by mosquitoes during the bites. This initiates the mosquito stages (gametes, ookinetes and oocysts). Oocysts are transmitted to the host, initiating the liver stage.

Postma et al. developed a novel desferrioxamine B (DFO) delivery system based on liposomes to treat malaria [113]. DFO is a siderophore that chelates ferric iron (iron is an vital component of red blood cells). Iron is an important nutrient for *P. vinckei* as it infects red blood cells. Contrasting to previous liposomal vaccine articles, the researchers investigated the lipid to drug composition and the bilayer fluidity effects on DFO delivery and protection from infection. The lipids were all anionic in charge due to the presence of egg PG. Three different treatments of DFO were analyzed in the study: (1) multiple free DFO subcutaneous injections, (2) intraperitoneal infusion of free DFO and (3) multiple subcutaneous DFO-loaded liposomes injections in C57B1/6J female mice. Parasitemia was suppressed by multiple subcutaneous injections of free DFO before and during infection, but injections prior to infection did not. Suppression of parasitemia and long-term survival was observed for intraperitoneal infusion of free DFO 1 day before infection or by subcutaneous injections of liposomal DFO prior to infection (day-1). Bilayer rigidity was studied by incorporating Chol (intermediate rigidity) and DSPC (high rigidity) in the liposomes, demonstrating that no relationship exists that affect the liposome antimalarial function. However, drug-to-lipid ratio affected the antimalarial activity of the liposomal-based vaccine, suggesting that low drug-to-lipid ratios are the best formulation parameter at combating the infection. When liposomal DFO was administered to mice, a long-term protection against malaria was observed (days 7 and 8, 400 mg/kg/day). This article proves that utilizing a common siderophore (DFO) as an iron chelator in combination with liposomes, improve the therapeutic and prophylactic effects of the vaccine. However, no immune response studies were performed, lacking essential information about the mechanisms of the vaccine when immunization occurs.

A pre-clinical report of the malaria vaccine RTS,S was published by Stewart et al. [114]. The RTS,S/AS02A vaccine utilizes the circumsporozoite protein as antigen. Investigators in this report evaluated the effects of certain adjuvants (AS01B, AS02A, AS05 and AS06) which vary in the concentration of MPL, QS21 or CpG and their formulation delivery system (emulsion vs. formulation). AS01B was the only liposomal formulation in the study. *Rhesus* macaques were immunized by intramuscular injection with the different RTS,S/adjuvant combinations and specific antibodies, IFN-γ and IL-5 levels were determined after weeks 14 and 34. All regimes were safe and presented elevated antibody titers (except for AS06-containing vaccine formulation). RTS,S/AS01B presented higher levels of IFN-γ at weeks 14 and 34, and the highest IFN-γ to IL-5 ratio when compared to RTS.S/AS02A. *Leishmaniasis.*

Another parasitic disease is leishmaniasis caused by the parasite *Leishmania* sp. It is transmitted by sandflies to mammals, transferring metacyclic promastigotes via feeding. The metacyclic promastigotes invade macrophages and granulocytes, developing into amastigotes which multiply by simple division, eventually causing macrophage lysis. Further, amastigotes infect new macrophages or are transferred to the sandflies during feeding. Amastigotes transform into procyclic promastigotes in the gut, maturing into metacyclic promastigotes by simple division. The disease is common in certain regions of Asia, Africa, South and Central America and even southern Europe. The disease is divided into three major syndromes, cutaneous, mucosal or visceral leishmaniasis. Visceral leishmaniasis poses a major risk of death incidence [115].

Three seminal articles provide valuable information regarding the studies and development efforts towards a prophylactic vaccine for leishmanial infections [116–118]. First, Bhowmick et al. presented the immunotherapy effects of leishmanial antigens in liposomes [116]. The researchers correlated the efficacy of soluble leishmanial antigens (SLAs) from *Leishmania donovani* promastigote membrane incorporated in neutral (lecithin:Chol), negative (lecithin:Chol:PA (phosphatidic acid)) and positively (lecithin:Chol:SA) charged liposomes intraperitoneally administered. *L. donovani* was eliminated from the liver and spleen when SLAs were present in cationic lipids. IL-4 and IL-10 were downregulated when SLA-cationic liposomes were administered to the mice and the immunomodulatory response presented the T_H_1 cytokines IFN-γ and IL-12. Subsequently, Banerjee et al. presented two articles which cover the study of cationic stearylamine liposomes for the development of a visceral leishmaniasis vaccine. In the first published article by the team of researchers, amphotericin B (AmB) is used in association with stearylamine (cationic) liposomes as a novel therapeutic approach [117]. When administered to BALBc mice, the leishmanial parasite was eliminated from the liver and spleen. Moreover, when comparing to the conventional liposomal formulation AmBisome, the intravenous administration of AmB-SA-PC liposomes induced the production of IFN-γ from CD8^+^ and CD4^+^ T cells. At the same time, the formulation reduced the toxicity effects of the drug by reducing TNF-α levels. In the splenic supernatant culture, IL-10 was downregulated, causing the production of IL-12 and nitric oxide during the AmB-SA-PC liposomes treatment. Also, Banerjee et al. investigated the effects of liposome charge in an antileishmanial assay [118]. Researchers provided evidence of membrane disruption caused by the cationic stearylamine liposomes in promastigotes and amastigotes. No toxicity in murine peritoneal macrophages and human erythrocytes was detected. These studies confirmed the prophylactic effect of SA-PC liposomes against leishmanial infections.

### Amoebiasis

Finally, a research paper dealing with parasitic infections present us the incorporation of *Entamoeba histolytica* Gal/GalNAc lectin LecA antigen in liposomal, emulsion and alum formulations containing synthetic TLR agonists adjuvants [59]. *E. histolytica* is an anaerobic amoeba and is the etiological agent of amoebiasis, or diarrheal disease commonly transmitted through contaminated water and food sources [119]. The liposome formulation containing a TLR4 and TLR7/8 agonists was selected for further studies due to its ability to induce intestinal IgA, plasma IgG2a/IgG1, IFN-γ and IL-17a. A high mucosal IgA response hinder the ability of parasites to adhere to mammalian cells. The subcutaneous immunization regime success rate reached 55% efficacy.

Parasitic infections are common especially in underdeveloped regions, posing a notable risk for children, adults and the elderly. Further studies should address the use of adjuvants and their immunomodulatory mechanisms when administered in liposomes for vaccine development. Several studies previously discussed optimized certain formulations for vaccine development and we invite the reader to have a look at them (Table 6). Additionally, specific antigens should be employed to induce even more specific immune responses that will enhance the eradication of relevant parasitic diseases like leishmaniasis and amoebiasis. Furthermore, studies should consider administration routes as potential factors that may affect the immunomodulatory responses of vaccines for the treatment of parasitic infections. For instance, toxicity of *L. donovani* was reduced when administered intravenously and differences in the immune response cytokine profile were detected. These cytokine profiles must need to be addressed, conducting studies with the similar liposomal formulation and administering the vaccines through different routes.

**Table 6:** Liposomal vaccine formulations tested in parasitic infection models

Lipid(s) and sterol used	Parasite	Cell line/animal model used	Administration route	Promising liposome formulation	References
MPL/QS21 (liposome-based)	*Plasmodium falciparum*	Rhesus macaques	Intramuscular	MPL/QS21 (liposome-based)	[114]
Egg lecithin/Chol, egg lecithin/SA and egg lecithin/PA	*Leishmania donovani*	BALB/c mice	Intraperitoneal	Lecithin/Chol/SA	[116]
PC/Chol, PC/SA, PC/PA and PC/PS	*L. donovani*	–	Intravenous, N/A	PC/SA	[117, 118]
EPC/EPG, EPC/EPG/Chol and DSPC/DPPG/Chol	*P. vinckei*	Female C57BL/6J mice	Intraperitoneal	EPC/EPG, EPC/EPG/Chol and DSPC/DPPG/Chol	[113]

Cell cultures and no vaccination in animal models were employed

*N/A* not applicable

## DDA-based cationic liposomal vaccines for the treatment of tuberculosis

The World Health Organization (WHO) estimated 1.8 million deaths in 2015 were related to tuberculosis infections (TBIs) [120]. The global estimate for latent tuberculosis infections (LTBI) was recently determined to be 23% (approximately 1.7 billion persons from the total population) [121]. Because of current tuberculosis (TB) vaccine efficacy variability, inefficiency and waning immunity (like Bacillus Calmette–Guerin, BCG) [57, 58], it is imperative to develop novel vaccines strategies that will improve prophylactic avenues for TBIs and reduce the overall death rate or latency associated with them. One of the principal improvements revealed in the last years is the development of adjuvanted subunit vaccines.

To diminish the detrimental effects of tuberculosis in humans, certain studies have focused on subunit vaccine development based in cationic lipid formulations (Table 7). Adjuvants are required for the development and efficacy of subunit vaccines, potentiating immune responses. Several adjuvants have been studied in the past 20 years including the adjuvanting properties of the lipid DDA [122]. Forty years ago, Snippe et al. investigated the effect of DDA in mice (via an intracutaneous administration) determining that delayed hypersensitivity occurred by the onset of footpad swelling 5 days after vaccination [123]. Subsequently, a study by van Houte et al., where low- (DOPC and DLPC) and high-transition temperature (DSPC) lipids were utilized with DDA as liposome bilayer components, discovered less immunogenicity of the liposomes as DDA concentration decreased [50]. Additionally, an earlier review discussed the immunostimulatory properties of DDA and its uses in different vaccines for veterinary and human infections [47]. We will present the reader with the most significant studies performed that cover the utilization of the cationic lipid DDA in subunit vaccine development.

**Table 7:** DDA-based TB vaccine formulation optimization studies

Lipid(s) and sterol used	Cell line/animal model used	Administration route	Promising liposome formulation	References
DDA, DDA/Tween 80, DDD/Span 85, DDA/Tween 80/Span 85, DDA/gelatin, DDA/Chol, DDA/Lecithin, DDA/β-Cyclodextrin and DDA/PLGA	C57BL/6 mice	Subcutaneous	DDA/Chol	[126]
DDA, DOTAP, DC-Chol, DOPE/PC and DOPE/PC/PG	Splenocytes from BALB/c and C57BL/6 mice	Subcutaneous	DDA	[125]
DDA/DSPC	Splenocytes from C57BL/6 mice	Intramuscular	DDA/DSPC	[18]
DDA	Splenocytes from BALB/c mice	Subcutaneous	DDA	[6]
DDA	Human/macrophage cell line THP-1 and splenocytes from BALB/c mice	Intramuscular	DDA	[30]
DDA	Inguinal lymph nodes or spleens	Subcutaneous	DDA	[128]
DDA	C57BL6; spleen and lung lymphocytes	Subcutaneous	DDA	[124]
DDA and DDA/Chol	Splenocytes from C57BL/6 mice and THP-1 cells	Intramuscular	DDA	[52]
DDA/MMG	Splenocytes from C57BL/6 mice	Subcutaneous	DDA/MMG	[134]
DDA	BALB/c mice	Intracutaneous	DDA	[122]
DDA	C57BL6 mice	Subcutaneous	DDA	[129]
DDA	BALB/c mice	Intracutaneous	DDA	[123]
DDA	Splenocytes from BALB/c mice	Intramuscular	DDA	[44]
LAM/PC/Chol/stearyl octaarginine	PBMCs	N/A	LAM/PC/Chol/stearyl octaarginine	[17]
DDA	DCs	Subcutaneous	DDA	[135]

Cell cultures and no vaccination in animal models were employed

*N/A* not applicable

Holten-Andersen et al. mixed the recombinant immunodominant *M. tuberculosis* antigens (ESAT-6 and Ag85B-ESAT6), DDA and different immunomodulators, analyzing the immune responses against BCG vaccination in mice (subcutaneous administration) [124]. The studied immunomodulators included saponin, calcitriol, β-glucan, *n*-hexadecane, TDB, muramyl dipeptide (MDP) and monophosphoryl lipid A (MPL). Investigators determined that the combination of the antigens with DDA and TDB generated a strong protective T_H_1 immune response against the mycobacterium, contrasting with BCG vaccination. In another study, *M. bovis* BCG lipid extracts were tested for their adjuvant characteristics [125]. BCG lipids were incorporated in DDA-based liposomes and administered subcutaneously to female BALB/c or C57BL/6 mice. BCG lipids coupled with the antigen Ag85B-ESAT-6 fusion protein in cationic liposomes induced significant levels of IFN-γ and relevant antibodies (IgG2A) titers, characteristic of T_H_1 immune responses. Antigens from other sources (*Chlamydia muridarum* and tetanus toxoid) were studied and relevant antibodies were detected when administered with the so called mycosomes (BCG lipids + cationic liposomes).

Subsequent studies on cationic liposomes bearing the antigen Ag85B-ESAT-6 (or its modifications) from *M. tuberculosis* were performed [6, 18, 52, 126]. Henriksen-Laceyet al. incorporated the antigen in DDA or DDA:TDB (8:1 molar ratio) liposomes and administered the vaccine formulation via intramuscular or subcutaneous injections to mice [6]. The antigen did not affect the liposome size, Zeta (ζ) potential or polydispersity index. The cationic lipid formulation was compared with antigen administered alone to mice. Investigators observed the rapid dissemination of the antigen administered alone in mice, contrasting with a depot formation at the injection site when administered in a liposomal formulation (up to 14 days post-vaccination). TDB allowed for the translocation of the liposomes form the site of injection to lymph nodes with the additional effect of monocyte infiltration to the injection site. Another study investigated the effects by which DDA plays a key role as an immunostimulatory lipid [18]. Mice were immunized intramuscularly with the proposed vaccine. Researchers concluded that removing or reducing DDA molar ratio in the liposome bilayer conduced to a reduction in T_H_1 immune responses against the antigen. Moreover, a team of researchers determined that the addition of cholesterol to the bilayer of DDA:TDB liposomes did not induced strong immune responses suggesting the prominent role of bilayer fluidity [52]. However, the previously mentioned study contrast with results obtained by Liu et al., were the TLR3 ligand Poly I:C was utilized as an adjuvant along with DDA and cholesterol liposomes (DPC liposomes) [126]. Researchers employed the TB fusion protein ESAT-6-Ag85B-MPT64(190-198)-Mtb8.4-Rv2626c (LT70) as the model antigen. DPC liposomes showed stability at size of 400 nm and ζ potential of 40 mV. Strong humoral and cell-mediated immune responses were detected by the production of antigen-specific antibodies after the subcutaneous administration and a markedly protection against a *M. tuberculosis* infection challenge than the traditional BCG vaccine.

With the advances in recombinant protein antigens from *M. tuberculosis*, other teams of researchers decided to observe at different antigenic proteins (like OVA) in DDA:TDB liposomes [44, 127], comparing different adjuvants and physicochemical properties in cationic liposomal formulations [30, 128] or investigating the mechanism of protein antigen adsorption [127]. OVA-containing SUVs liposomes composed of DDA:TDB with no TLR ligand showed a higher capacity to induce spleen CD8 IFN-γ responses against the antigen, contrasting to MLVs, administered intramuscularly [44]. Antigen-specific responses were higher on SUVs. Adding TLR3 and TLR9 agonists significantly increased the immune responses on MLVs carrying OVA, but that was not observed in SUVs. The study suggested that liposomes are an excellent delivery vehicle for antigen presentation and vaccine formulation, and we believe that this is the foundation for the subsequent studies based on cationic and neutral lipid formulations. Investigators selected DDA and the synthetic mycobacterial cord factor molecule, TDB, as a suitable model adjuvant (CAF01) for vaccine development [128]. This was based in that mice vaccination (subcutaneous) with CAF01 induced a strong antigen-specific cell- and humoral-mediated responses, contrasting with currently used adjuvants (e.g. alum and MPL). Furthermore, results were strongly supported by the protection from infection when mice were challenged by *M. tuberculosis*, *C. trachomatis* or malaria, revealing cell mediated (TB), cell-mediated/humoral (*C. trachomatis*) and humoral immune responses.

A study showed the significant role that liposome vesicle size plays in the cell-mediated immune responses [30]. Researchers determined that no differences in vaccine (administered intramuscularly) draining from the injection site occurred, but a size-dependent liposome movement (favored by large liposomes) was observed to the popliteal lymph node. Macrophage-like cells internalized liposomes in a size-independent pattern. Size did not affect the antigen-specific antibody response (IgG1/2) of Ag85B-ESAT-6-carrying liposomes, however larger liposomes induced highest cell proliferation and lowest IL-10 levels, which contrasted with smaller vesicles (inducing IFN-γ and IL-1β). Additionally, a study investigated the effects of charge, membrane fluidity and antigen-to-lipid ration on mechanism of protein antigen adsorption [127]. For this purpose, investigators compared cationic (CAF01) and neutral (NAF01) liposomal formulations, mainly composed of DDA or DSPC, respectively. α-Lactalbumin and lysozyme were used as antigen protein models to analyze the parameters. The anionic lactalbumin interacted with cationic liposomes by surface adsorption, and no interaction was observed with zwitterionic liposomes. However, the cationic lysozyme presented no detectable interaction with either type of liposomes. Adsorption of α-lactalbumin generated changes in its tertiary structure, neutralized liposome charge, affected lipid membrane packing (especially for CAF01 liposomes), resulting in a reduction of colloidal stability and liposome aggregation. The CAF01 formulation has completed multiple phase I safety trails in humans to date.

Other studies have focus their attention on cell components, synthetic lipid analogues or mycobacterial lipids as antigens for subunit vaccine development employing DDA as the principal component in the lipid bilayer. Mutant *M. bovis* BCG *ΔmmaA4* strain was formulated in cationic liposomes of DDA:TDB (A4/Adj) and administered subcutaneously [129]. The mutation deletes the *mmaA4* gene that encodes a *S*-adenosylmethionine-dependent methyltransferase involved in mycolic acid biosynthesis in the tubercle bacillus [130]. Immunocompromised mice (TCRδ^−/−^) immunized with A4/Adj were protected against an infection of *M. tuberculosis* (2 and 9 months post-vaccination), contrasting with non-adjuvanted mutant and non-vaccinated controls. It is important to note that the immunocompromised mice lack CD4^+^, CD8^+^ and NK1.1^+^ T cells but, due to immunization long-term results, an unconventional T cell population was responsible for the immune responses. Researchers observed CD4^−^ CD8^−^ double negative (DN) T cells and found that the cells accumulated in the lungs of A4/Adj-treated mice, with significant levels of IFN-γ production when comparing to nonvaccinated or nonadjuvanted BCG control test groups. In vitro studies revealed the antimycobacterial properties of DN T cells isolated from adjuvanted BCG-treated mice when compared to whole-spleen cells. The results of this study represent a milestone in medical research since tuberculosis affects dramatically immunocompromised patients, like in HIV infections [131–133].

Synthetic lipid analogues from monomycoloyl glycerol (MMG-1 to 6) from *M. tuberculosis* has been developed and their supramolecular structure and adjuvant efficacy tested via subcutaneous administration [134]. The analogues displayed longer (MMG-2) or shorter (MMG-3) alkyl chains, or stoichiometry variations of the polar head group (MMG-5) or the hydrophobic moiety (MMG-6). CryoTEM and synchrotron small-angle X-ray (SAX) experiments revealed the supramolecular organization varied from unilamellar and multilamellar (ULVs/MLVs) vesicles in DDA:MMG-1/2/5/6 liposomes to ULVs and hexosomes in DDA:MMG-3. T_H_1 and T_H_17 immune responses were induced by DDA:MMG-1/3/6 liposomal formulations in response to a chlamydial antigen, contrasting to different immunostimulatory properties of naked MMG-1 and MMG-6 analogues in vitro. We recommend further studies employing MMG analogues incorporated in liposomes with mycobacterial-derived antigens to assess the efficacy of this chlamydial model. In contrast, another study utilized natural mycobacterial lipids diacylated sulfoglycolipids (Ac_2_-SGL) and phosphatidyl-*myo*-inositol dimannosides (PIM_2_) as antigens in a liposomal vaccine formulated with DDA and TDB as adjuvants [135]. Researchers observed a reduction of bacterial load in the spleen of vaccinated animals via subcutaneous administration, contrasting with the unvaccinated group. The lipid antigen vaccine group showed a remarkable reduction of lung and spleen lesions when compared to the unvaccinated group. Comparison of lipid antigen vaccine with protein antigen vaccine regimes in a guinea pig model revealed no significant differences in the treatments.

From the articles discussed in this section we can count on promising advances in tuberculosis vaccine development. The approaches presented applied recombinant protein antigens, whole-mutant cell and synthetic and natural cell components formulations. Each approach could encounter some drawbacks: mass production of natural and synthetic products from *M. tuberculosis* and other mycobacteria; liposomal formulation instability and aggregation; unwanted immune responses. Most of the studies discussed above demonstrated that subcutaneous administration could be a successful route of administration and future research should direct efforts to determined other administration routes, like intranasal and intramuscular. By understanding the physicochemical parameters of liposomes and the effects of routes of administration on physiological and immune function requirements, scientists will be able to develop a novel tuberculosis vaccine.

## Conclusion

Vaccination represents the major advancement of modern medicine, second in effect only to clean water, in decreasing the spread of detrimental diseases and providing better quality of life. To develop effective and safe vaccines, collaboration between immunologists and formulation scientists must exist. This collaboration will ensure that the vaccine is designed adequately and that the therapy will not cause unwanted immune responses. Also, it is important to understand the basic concepts in immunology (innate vs. adaptive immunity) so we can target the corresponding receptors with the ligands and antigens employed in the vaccine. Many types of cell receptors participate in PAMPs recognition (innate immunity), namely NLRs, STING, RLRs, TLRs and CLRs. Each receptor contributes to significant responses that lead to T helper cell activation and differentiation, with eventual B cell (antibody-mediated) and CD8 T cell-mediated adaptive immune responses.

For liposomal vaccines, we must pay close attention to liposome size, surface charge (ζ potential), morphology (lamellarity) and lipid bilayer fluidity. Size may affect antigen presentation to APCs and consequently immune responses. Likewise, surface charge of the liposome may affect antigen adsorption on the liposome and could affect liposome-cell interactions (cationic liposomes interact better with the anionic membrane of immune cells). MLVs may reduce antigen presentation due to their concentric vesicle morphological nature, contrasting to ULVs which enhances antigen presentation. Antigen leakage and immune response reduction may occur on how the antigen was formulated (adsorption vs. absorption). Absortion is explained by the T_m_ of the lipids or phospholipids used when developing the vaccine. Lipids with lower T_m_ tend to be fluid meanwhile lipids with higher T_m_ would increase bilayer rigidity. During adsorption, the antigen is exposed in the outer layer of the lipid membrane, which could allow easy release or presentation of the antigen. Additionally, we must pay special attention to how liposomes would interact with immune cells and how we can target those cells, enhancing proper immune responses. Adjuvants play a significant role in APCs activation and maturation for eventual T and B cell activation. The adjuvants, properly selected, will interact with their corresponding TLRs or CLRs in a process called targeted cell vaccine delivery. This cell targeted process is also affected by the vaccine route of administration.

We observed in the sections previously discussed key developments in contemporary vaccine research for the treatment of viral, bacterial, fungal and parasitic infections. The studies presented to the reader demonstrated the collaborative and interdisciplinary nature of the field. Cell targeting and adjuvants dominated most of the approaches to develop effective prophylactic subunit vaccines for the treatment of detrimental diseases. In some instances, infection was hindered by the antimicrobial, antiviral, antifungal or antiparasitic activities of the vaccines due to the induction of adequate immune responses (cell- and antibody-mediated), leading to the survival of selected animal models. Additionally, we can conclude that cationic liposomal vaccines are of great interest for future vaccine development due to their enhanced interaction with the negatively charged immune cells. In specific, DDA-based liposomes are being tested in diverse vaccine studies which promise significant advances in the field. Such DDA applications as a building block of liposomal vaccines represent a step forward towards the prophylactic treatment of diverse infections. These developments will improve the current vaccine approaches and will provide better treatments for patients. Additional research efforts must be made towards the development of novel adjuvants that will contribute to the induction of significant immune responses. Both authors read and approved the final manunscript.

## Abbreviations

AmB: amphotericin B
APCs: antigen presenting cells
BCG: Bacillus Calmette–Guerin
BCR: B cell receptor
BMDCs: bone marrow dendritic cells
BSA: bovine serum albumin
CCS: ceramide carbamoyl-spermine
Chol: cholesterol
CLR: C-type lectin receptor
COPD: chronic obstructive pulmonary disease
CpG-ODN: CpG-containing oligodeoxynucleotides
CWSPs: cell wall surface proteins
DAMPs: damaged-associated molecular patterns
DCs: dendritic cells
DCC: *N,N*-dicyclohexylcarbodimide
DC-Chol: dimethylaminoethane-carbamoyl-cholesterol
DC-SIGN: DC-specific ICAM3-grabbing non-integrin
DDA: dimethyldioctadecylammonium
Dectin-1: dendritic cell-associated C-type lectin 1
Dectin-2: dendritic cell-associated C-type lectin 2
DFO or DFB: desferrioxamine B
DLPC: 1,2-dilauroyl-sn-glycero-3-phosphocholine
DMPC: 1,2-dimyristoyl-sn-glycero-3-phosphocholine
DMPG: 1,2-dimyristoyl-sn-glycero-3-phospho-(1ʹ-rac-glycerol)
DMTAP: 1,2-dimyristoyl-3-trimethylammonium-propane
DOGS-NTS-Ni: 1,2-dioleoyl-sn-glycero-3-[(*N*-(5-amino-1-carboxypentyl)iminodiacetic acid)succinyl] (nickel salt)
DOPC: 1,2-dioleoyl-sn-glycero-3-phosphocholine
DOPE: 1,2-dioleoyl-sn-glycero-3-phosphoethanolamine
DOTAP: 1,2-dioleoyl-3-trimethylammonium-propane
DOTMA: 1,2-di-*O*-octadecenyl-3-trimethylammonium propane
DPPC: 1,2-dipalmitoyl-sn-glycero-3-phosphocholine
DSPC: distearoyl-sn-glycero-3-phosphocholine
DSTAP: 1,2-stearoyl-3-trimethylammonium-propane
DTA: diphtheria toxin
EPC: egg phosphatidylcholine
HBsAg: hepatitis B surface antigen
HBV: hepatitis B virus
HIV: human immunodeficiency virus
Hsp90: *C. albicans* heat shock protein 90
IFN-γ: gamma interferon
Ig: immunoglobulin
IL: interleukins
i.n.: intranasal
LAg: leishmanial antigen
Lip: liposome(s)
LPS: lipopolysaccharide
LTBIs: latent tuberculosis infections
LUVs: large unilamellar vesicles
ManLAM: mannose lipoarabinomannan
Man-PE: mannosyl phosphatidylethanolamine
MCL: macrophage C-type lectin
MDDCs: monocyte-derived dendritic cells
MDMs: monocyte-derived macrophages
MDP: muramyl dipeptide
MHC I: class I major histocompatibility complex
MHC II: class II major histocompatibility complex
MINCLE: macrophage-inducible C-type lectin
MLVs: multilamellar vesicles
MMG: monomycoloyl glycerol
MO: monooleoylglycerol
MPL or MPLA: monophosphoryl lipid A
MR: mannose receptor
NKs: natural killer cells
NLRs: nucleotide-binding domain and leucine-rich repeat receptor
OVA: ovabulmin
PA: phosphatidic acid
PAMPs: pathogen associated molecular patterns
PC: phosphatidylcholine
PG: phosphatidylglycerol
PMNs: polymorpho nuclear cells
POPC: 1-palmitoyl-2-oleoyl-sn-glycero-3-phosphocholine
POPE: 1-palmitoyl-2-oleoyl-sn-glycero-3-phosphoethanolamine
PRRs: pathogen recognition receptors
PS: phosphatidylserine
RLRS: retinoic acid-inducible gene I receptor
RSV: respiratory syncytial virus
SA: stearylamine
SDC: sodium deoxycholate
SUVs: small unilamellar vesicles
TAMPs: tumor-associated molecular patters
TB: tuberculosis
TBIs: tuberculosis infections
TCR: T cell receptor
TDB: trehalose dibehenate
T_H_ cells: T helper cells
TLR: toll-like receptor
TNF-α: tumor necrosis factor alpha
WHO: World Health Organization
